# Supplementary material for: P-selectin mobility undergoes a sol-gel transition as it diffuses from exocytosis sites into the cell membrane
Source: Nat Commun. 2022 May 31;13:3031. doi: 10.1038/s41467-022-30669-x (PMC9156680; doi:10.1038/s41467-022-30669-x)
Supplement: Supplementary file 1 — Supplementary Information [file 41467_2022_30669_MOESM1_ESM.pdf]

Supplementary Tables and Figures

Hellen *et al.* ***"****P-selectin mobility undergoes a sol-gel transition as it diffuses from exocytosis sites across the endothelial cell plasma membrane."*

Supplementary Tables 1 and 2

Supplementary Figures 1-13

References

Supplementary Table 1.

Antibody reagents and Validation

	Species	Manufacturer	Clone / catalogue number	Dilutions used for ICC or WB
**1) VWF**	Rabbit pAb	DAKO/Agilent<br>Santa Clara, CA USA	A0082	1:10000
**2) VWF**	Sheep pAb	Serotec<br>Raleigh, NC, USA	AHP062	1:10000
**3) P-selectin**	Mouse pAb	Bio-Rad (formerly AbD Serotec)	AK6/MCA796	1:50
**4) Control mouse IgG**	Mouse pAb	Invitrogen/ThermoFisher	16-4714-82	1:50
**5) Heparan sulphate**	Rabbit mAb	Sigma-Aldrich	MAB1948P/A7L6	1:500
**6) Chondroitin-4-sulphate**	Mouse mAb	Sigma-Aldrich	MAB2030/clone BE-123	1:50
**7) FITC-WGA**	-	Vector Labs Inc<br>Burlingame, CA, USA	FL-1021	1:400
**8) anti-GFP**	Rabbit mAb	Invitrogen/ThermoFisher	A-6455	1:300
**9) anti-GFP**	Sheep pAb	Bio-Rad Labs<br>Watford, UK	4745-1051	1:250
**10) AP2alpha**	Mouse mAb	Invitrogen/ThermoFisher	AP6/ MA1-064	1:400
**11) anti RFP**	Rabbit pAb	Abcam<br>Cambridge, UK	ab62341	1:200
**12) Rhodamine-phalloidin**	-	Invitrogen/ThermoFisher	R415	1:200
**13) GROα**	Mouse MAb	R&D systems<br>Abingdon, UK	MAB275 / clone 20326	1:100
**14) IL-8**	Goat pAb	R&D systems	AF-208-NA	1:100
**15) Anti-alpha adaptin**	Mouse mAb	BD Transduction labs	610502	1:1000 (WB)
**16) GAPDH (control : 36kDa)**	Mouse mAb	Merck Millipore	MAB374, clone 6C5	1:5000 (WB)
**17) Fluorophore coupled 2^0**	various	Jackson ImmunoResearch Europe (Newmarket, UK)	**Alexa 488** AffiniPure Donkey Anti-Mouse IgG (715-545-150)<br>**Rhodamine Red™-X** (RRX) AffiniPure Donkey Anti-Mouse IgG (115-295-003)<br>**Cy™5** AffiniPure Donkey Anti-Rabbit IgG (711-175-152)<br>**Alexa 488** AffiniPure Donkey Anti-Rabbit IgG (711-545-152)<br>**Rhodamine Red™-X** (RRX) AffiniPure Donkey Anti-Rabbit IgG (711-295-152)<br>**Alexa 488** AffiniPure Donkey Anti-Sheep IgG (713-545-147)	1:1000

Validation key (numbers as in Supplementary Table 1).

1. **Anti-Human VWF (A0082)** has been validated for detection of von Willebrand factor in endothelial cells, megakaryocytes and platelets when tested on formalin-fixed, paraffin-embedded normal human tissues (bone marrow, kidney, liver, lung, lymph node, skin and spleen) as described on the Agilent website https://www.agilent.com/en/product/dako-omnis-solution-for-ihc-ish/primary-

antibodies-for-dako-omnis/primary-antibodies-(flex-ready-to-use)/von-willebrand-factor-(dako-omnis)-76216). We use the Ab extensively in our lab for ICC and WB and it recognises epitope tagged VWF both in fixed and live HUVEC.

2. **Anti-human VWF** Ab has been verified for detection of VWF by Serotec and in multiple publications as described on the BioRad website https://www.citeab.com/antibodies/111902-ahp062-sheep-anti-human-von-willebrand-factor). We have used the Ab extensively in our lab for ICC and WPB and it recognises epitope tagged VWF in fixed aHUVEC.
3. **Anti-CD62P/P-selectin (AK6)**, Immunofluorescence; has been verified for detection of P-selectin by Serotec labs and in multiple publications as described on the BioRad website (https://www.bio-rad-antibodies.com/monoclonal/human-cd62p-antibody-ak-6-mca796.html?f=purified). In our hands AK6 recognises epitope tagged P-selectin expressed in HUVEC.
4. **Control mouse IgG**: This is commonly used as an isotype control reagent as described on the manufactures website (https://www.thermofisher.com/antibody/product/Mouse-IgG1-kappa-clone-P3-6-2-8-1-Isotype-Control/16-4714-82). Cy3b conjugated IgG was used on 3% paraformaldehyde fixed and live HUVEC and showed no labelling of cells when probed with a goat anti-mouse secondary Ab from Jackson ImmunoResearch.
5. **Anti-Heparan sulphate Ab (MAB1948P)** this Anti-Heparan Sulfate Proteoglycan (Perlecan) Antibody, clone A7L6, is validated for use in IH, IC, WB & IP according the manufactures web site https://www.merckmillipore.com/GB/en/product/Anti-Heparan-Sulfate-Proteoglycan-Perlecan-Antibody-clone-A7L6,MM_NF-MAB1948P .
6. **Anti-Chondroitin-4-sulphate (MAB2030)**: Immunofluorescence; this antibody only reacts with digested material. Fixed tissue must be digested with chondroitinase ABC (Reine, TM., et al., PubMed ID: 23757342). No reaction with native proteoglycans or with proteoglycans digested with either chondroitinase AC or testicular hyaluronidase. Reacts with both mouse (fetal and adult) and human (fetal and adult) chondroitinase ABC digested proteoglycans. https://www.sigmaaldrich.com/GB/en/product/mm/mab2030 .
7. **FITC-WGA (FL-1021)**: Immunofluorescence; detects N-Acetylglucosamine, positive staining validated by pre-blocking the lectin with its inhibiting sugar Chitin Hydrolysate. Used in >130 publications according to manufactures website https://vectorlabs.com/products/glycobiology/fluorescein-wheat-germ-agglutinin-wga#biozbadges.
8. **Anti-GFP rabbit (A-6455)**: Immunofluorescence, WB. Validated by WB in HEK293 cells expressing GFP (manufactures website). Detected GFP-tagged P-selectin expressed in HUVEC. More than 120 publications using the Ab for detection of GFP by Immunofluorescence (https://www.thermofisher.com/antibody/product/GFP-Antibody-Polyclonal/A-6455)
9. **Anti-GFP sheep (4751-1051)**: Immunofluorescence, this product has been reported to work by Immunofluorescence from testing within Serotec laboratories, and in multiple peer-reviewed publications, as described on the suppliers web site https://www.bio-rad-antibodies.com/polyclonal/green-fluorescent-protein-antibody-4745-1051.html?f=purified. In our hands it recognised GFP-tagged P-selectin expressed in HUVEC.
10. **Anti-AP2alpha, MA1-064**. Detects assembly polypeptide 2 (AP2) from a wide variety of mammalian sources, including human, hamster, monkey, bovine, rat, and mouse. It recognizes the products of both alpha-adaptin genes, alpha A and alpha C as well as an alternatively spliced isoform of alpha adaptin found in neurons. MA1-064 has been successfully used in Western blot, immunofluorescence, immunocytochemistry, and immunoprecipitation procedures. By Western blot, this antibody detects an ~100 kDa doublet representing the two isoforms of alpha adaptin in rat brain (alpha C is the dominant form seen).

11. **Anti-RFP antibody : ab62341** recognizes RFP and has been shown to react with tdTomato, It is validated for ICC and a wide range of other techniques. https://www.abcam.com/rfp-antibody-ab62341.html?productWallTab=Abreviews&applications=3688&PageSize=10&SortOrder=VoteDesc .
12. **Rhodamine-phalloidin (R415).** Rhodamine phalloidin is one of the most commonly used fluorescent phalloidin conjugates in the literature, as evidenced by over 1,500 citations https://www.thermofisher.com/order/catalog/product/R415
13. **Anti-GROα (MB275).** Validated for immunofluorescence in our laboratory as described in Knipe, L. *et al. Blood* **116**, 2183-2191 (2010).
14. **Anti-IL-8 (AF-208-NA).** Validated for immunofluorescence by the manufacturer and used successfully in our laboratory as described in Knipe, L. *et al. Blood* **116**, 2183-2191 (2010).
15. **Anti-alpha adaptin (PA5-17029).** This antibody is validated and routinely tested by Western blot by the manufacturer as described on their web pages https://www.bdbiosciences.com/ja-jp/products/reagents/microscopy-imaging-reagents/immunofluorescence-reagents/purified-mouse-anti-adaptin.610502
16. **Anti-GAPDH:** clone 6C5 is a well published and extensively characterized monoclonal antibody. This purified mAb detects Glyceraldehyde-3-Phosphate Dehydrogenase (GAPDH) & has been published & validated for use in ELISA, IP, IC, IF, IH & WB.
17. **Fluorophore-coupled secondary antibodies**: These are well-established lab reagents principally validated by the same methods as primary Abs as well as negative controls lacking primary antibodies (see e.g. above). They have been independently validated by the manufacturer, our own laboratories and many labs around the world.

Supplementary Table 2.

Primers used for cloning and mutagenesis

Primer	Sequence
P selectin ΔCTLD-f	5′ CGAGCTGATATCTACACAGCCTCCTGCCAG 3′
P selectin ΔCTLD-r	5′ GCCAGCCGATATCACGGGAAATATTCCATG 3′
P selectin ΔCTLD EGF-f	5′ CCGTGGGATATCTACGTGAGAGAGTGTGGAG 3′
P selectin ΔCTLD EGF-r	5′ GCCAGCCGATATCACGGGAAATATTCCATG 3′
P-selectin-Δ4CR-f	5′ ACGATATCTTGCAGTGCCAGGATCTCCC 3′
P-selectin-Δ4CR-r	5′ ACGATATCCACGTATTCACATTCTGGCCC 3′
P-selectin-Δ8CR-f	5′ TGGATATCGTGAAATGCTCAGAACTACATGTTAATAAG 3′
P-selectin-Δ8CR-r	5′ ACGATATCCACGTATTCACATTCTGGCCC 3′
P selectin ΔCT-f	5′ CAATTCATCTGTGACGAGGG 3′
P selectin ΔCT-r	5′ GCAAAGTCTGTTTTTCTACTACCCAGCTGATA 3′

Supplementary Figure 1

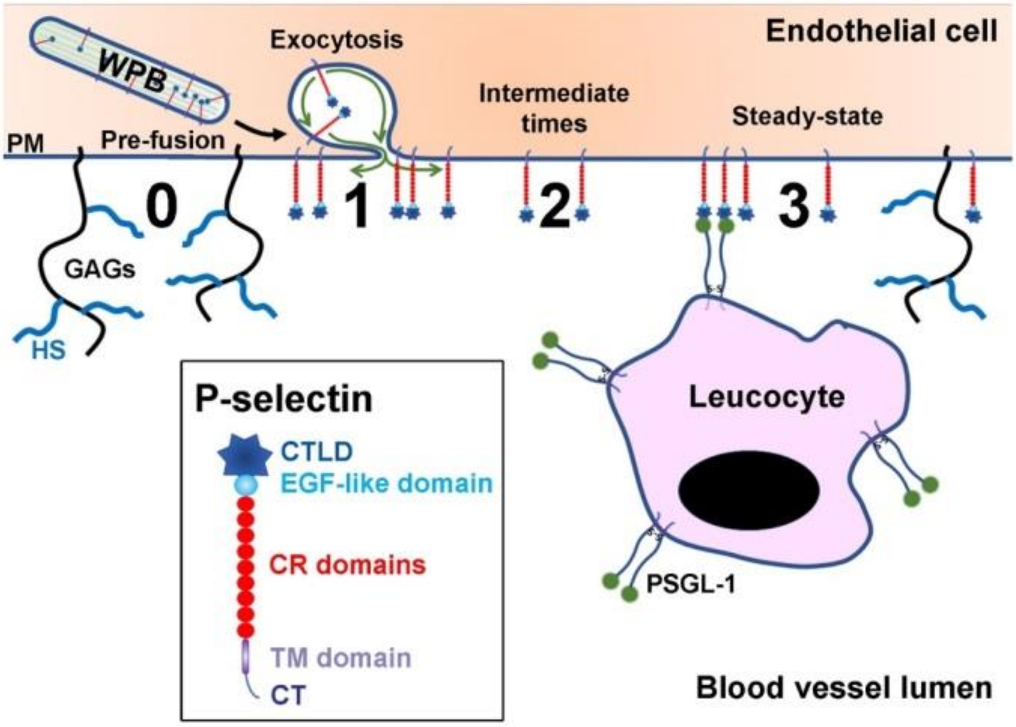

Supplementary Figure 1. **P-selectin secretion and function**. **0**) Under resting conditions, P-selectin is stored in an immobile state inside Weibel-Palade bodies (WPBs) and little or no P-selectin is present on the endothelial plasma membrane. **1**) Following endothelial activation by injury or infection, WPBs undergo exocytosis, P-selectin becomes mobile inside the post-fusion WPB and begins to diffuse into the plasma membrane. **2**) Immediately after exocytosis P-selectin continues to diffuse and spread into the plasma membrane. **3**) At later times (steady-state), clusters appear that are thought to facilitate adhesion and rolling of leucocytes on the surface of the blood vessel. Heparan sulphate containing glyco-proteins and glyco-lipids from part of the complex extracellular assemblies through which P-selectin must diffuse once in the plasma membrane.

Supplementary Figure 2

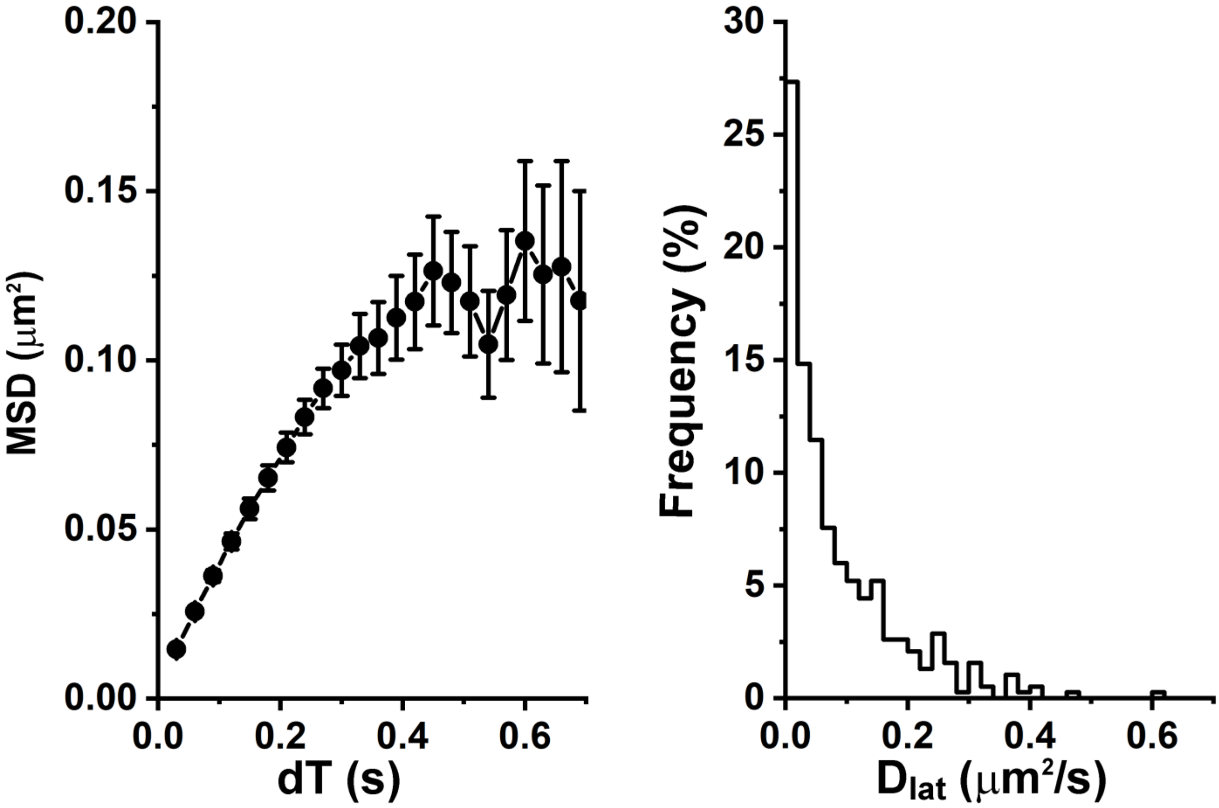

Supplementary Figure 2. Pre-stimulation, P-selectin-eGFP shows highly restricted mobility. *MSD* vs *dT* plots (**left**), dots showing the mean *MSD* value at each given *dT* and vertical bars delimit +/- s.e.m. and distributions of D_{lat} (**right**) for WT P-selectin-eGFP on the plasma membrane in HUVEC 0-48 s prior to cell stimulation (387 SMs, n=12 cells, 2 experiments). The mobile fraction (defined as molecules with D_{lat}> 0.05 $\mu m^2.s^{-1}$) was 54 ± 2.3%. Note the strong downward curvature of the *MSD* vs *dT* plot indicating highly-restricted diffusion. Non-linear, least-squares, fitting of the data to Main Text Equation 1 gave D_{lat} = 0.166 ± 0.013 $\mu m^2.s^{-1}$ and limiting $\overline{MSD}$ = 0.120 ± 0.013 μm^2.

Supplementary Figure 3

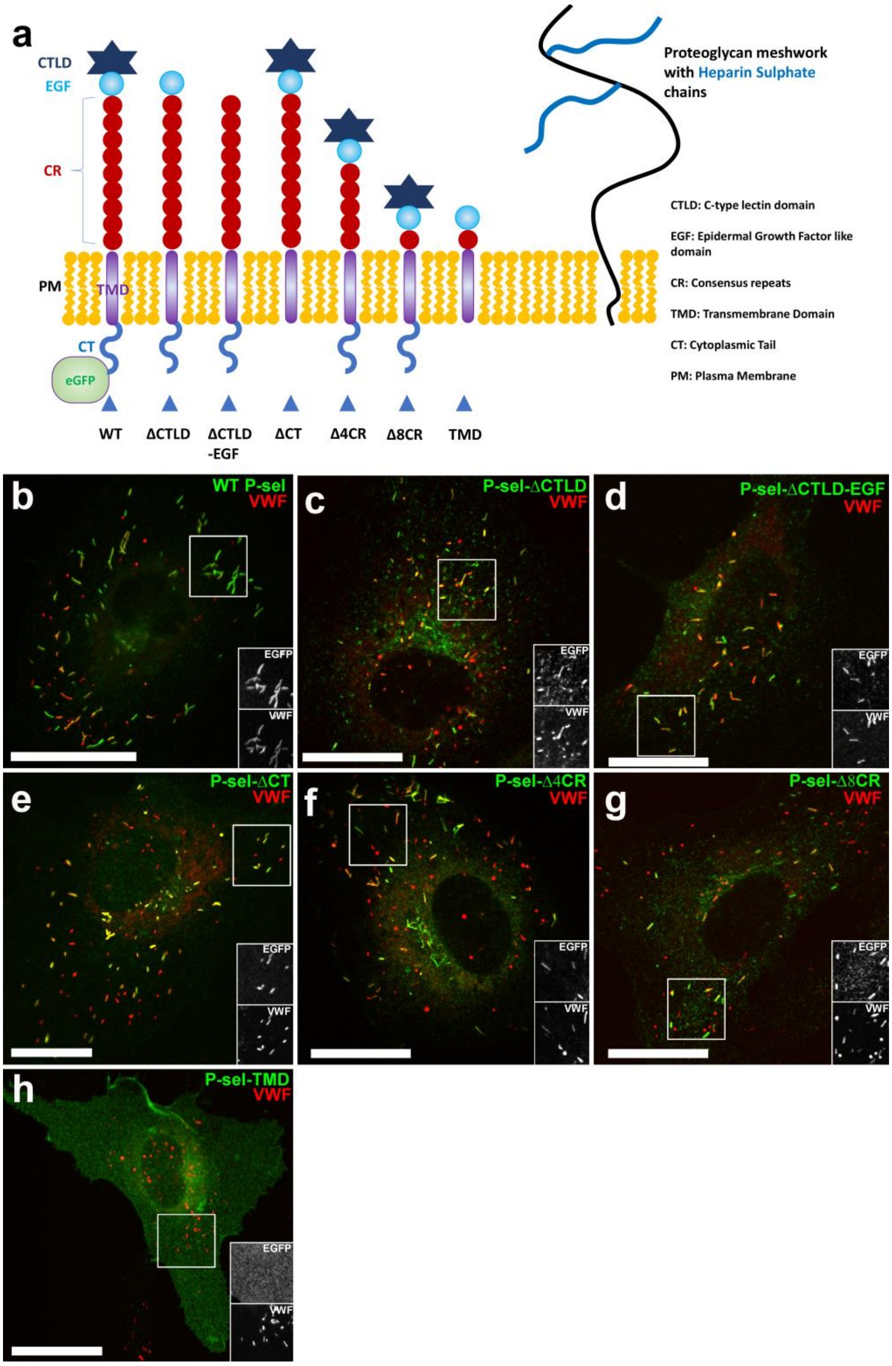

Supplementary Figure 3. P-selectin mutants. **a**) Cartoon depicting the deletion mutants used in this study. **b-h**) Confocal fluorescence images of fixed HUVEC 24-48 h after nucleofection with WT P-selectin-eGFP or P-selectin deletion mutants as indicated. Cells were immuno-labeled for eGFP (green; mouse monoclonal, clone 7.1) and endogenous VWF (red; sheep anti-human VWF antibody). Regions indicated by the white boxes are shown inset in grey scale, and show localization of eGFP to WPBs. Regions indicated by the white boxes are shown inset in grey scale, and show localization of eGFP to WPBs in all cases except TMD. Images are representative of 3 independent experiments. Scale bars 20 µm.

Supplementary Figure 4

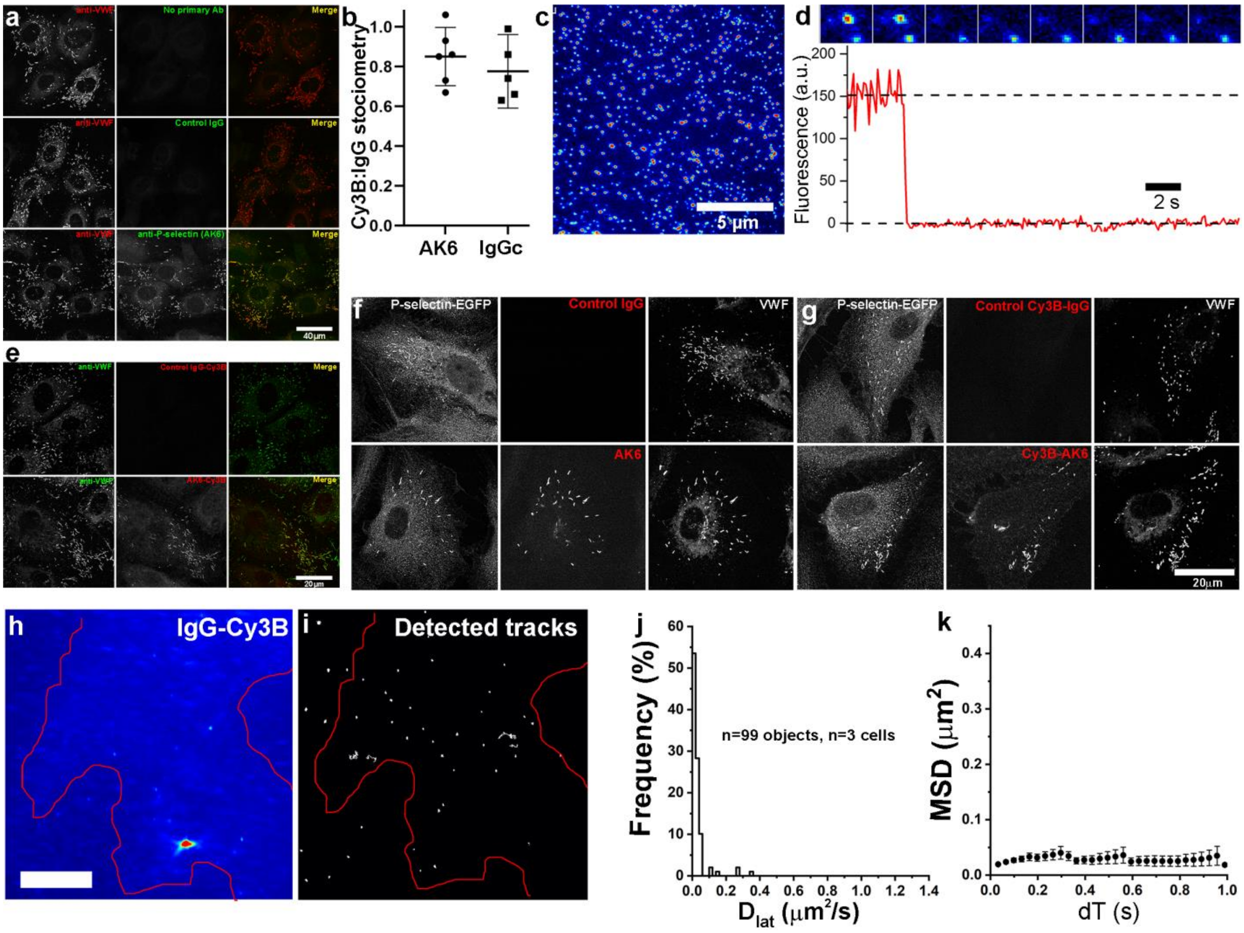

Supplementary Figure 4. Labeling of the anti-P-selectin antibody AK6 with Cy3B does not interfere with detection or trafficking of P-selectin in live HUVEC. a) Confocal fluorescence images of HUVEC immuno-labeled for endogenous von Willebrand Factor, VWF, alone (top panels; red; sheep anti-human VWF, antibody), VWF in the presence of isotype control mouse IgG (middle panels IgG; green), or the anti-P-selectin antibody AK6 (bottom panels, AKG; green). Scale bar, 40 µm. **b**) shows the measured stoichiometry of AK6 IgG labeling with Cy3B determined as described in methods (central horizontal bar is mean, Whiskers are 5-95% data range; n=6 replicates for Cy3B AK6, n=5 replicates control IgG, all data shown). **c**) TIRFM image of AK6-Cy3B (1:50,000 dilution) bound to a glass coverslip in phosphate buffered saline. **d**) Typical single-step photobleaching time-course of AK6-Cy3B fluorophore during continuous illumination with 561 nm laser light. **e**) Confocal fluorescence images of HUVEC immuno-labeled for endogenous VWF (green in merged image) and co-labeled with either control mouse IgG-Cy3B (top panel; red in merged image) or AK6-Cy3B (bottom panel, red in merged image). Images are representative of 5 independent experiments; Scale bar = 20 µm. **f&g)** Confocal fluorescence images of P-selectin-eGFP expressing HUVEC co-immuno-labeled for eGFP (left panels; sheep anti-GFP) and endogenous VWF (right panels; rabbit anti-human VWF). Prior to fixation and immuno-labeling live P-selectin-eGFP transfected cells were incubated for 24 h with either control mouse IgG (**f** top middle panel), AK6 (**f** bottom middle panel)**,**

control mouse IgG-Cy3B (**g** top middle panel) or AK6-Cy3B (**g** bottom middle panel). Images are representative of 4 independent experiments, Scale bars, 20 µm. **h)** Representative TIRFM image of the footprint of live HUVEC (marked by red perimeter lines on the image) after incubation with control mouse IgG-Cy3B (1:25 dilution, 30 min room temperature). Images are representative of 2 independent experiments. Scale bar 10 µm. **i)** shows the x-y trajectories (white tracks) of detected Cy3B SFs in the cell in **h.** **j-k)** show the distribution of D_{lat} (**j**) and *MSD* vs *dT* plot (**k**) for control IgG-C3B SFs (n=99 SFs, n=3 cells).

Supplementary Figure 5

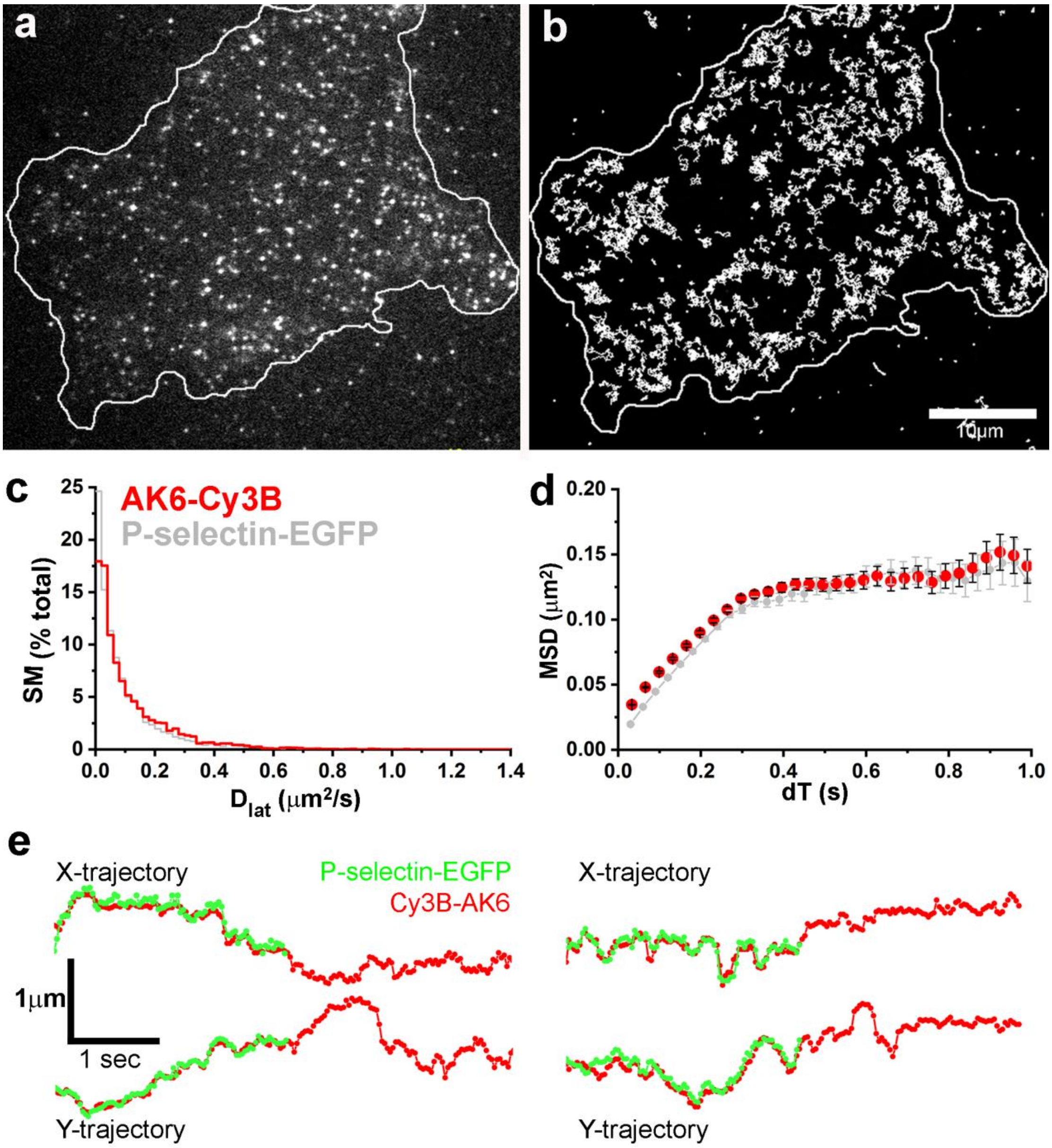

Supplementary Figure 5. AK6-C3B labeling of endogenous P-selectin does not perturb P-selectin mobility. **a**) TIRFM image of the footprint of live HUVEC (marked by white perimeter lines on the image) after a 30 min incubation with AK6-Cy3B. **b**) x-y trajectories of detected SFs in this cell (white tracks). **c)** Distribution of D_{lat} and **d**) *MSD* vs *dT* plot (as described for Supplementary Figure 2) for AK6-Cy3B (4,050 single molecules, n=4 cells, 3 experiments). For reference steady-state data for WT P-selectin-eGFP is shown in grey (from Figure 2 Main Text). **e**) Examples of co-incident x-y trajectories of individual objects containing both a single Cy3B (red) and eGFP (green) fluorophore on the plasma membrane of P-selectin-eGFP expressing HUVEC incubated with AK6-Cy3B. Note that the eGFP (green) traces terminate when the fluorophore bleaches.

Supplementary Figure 6

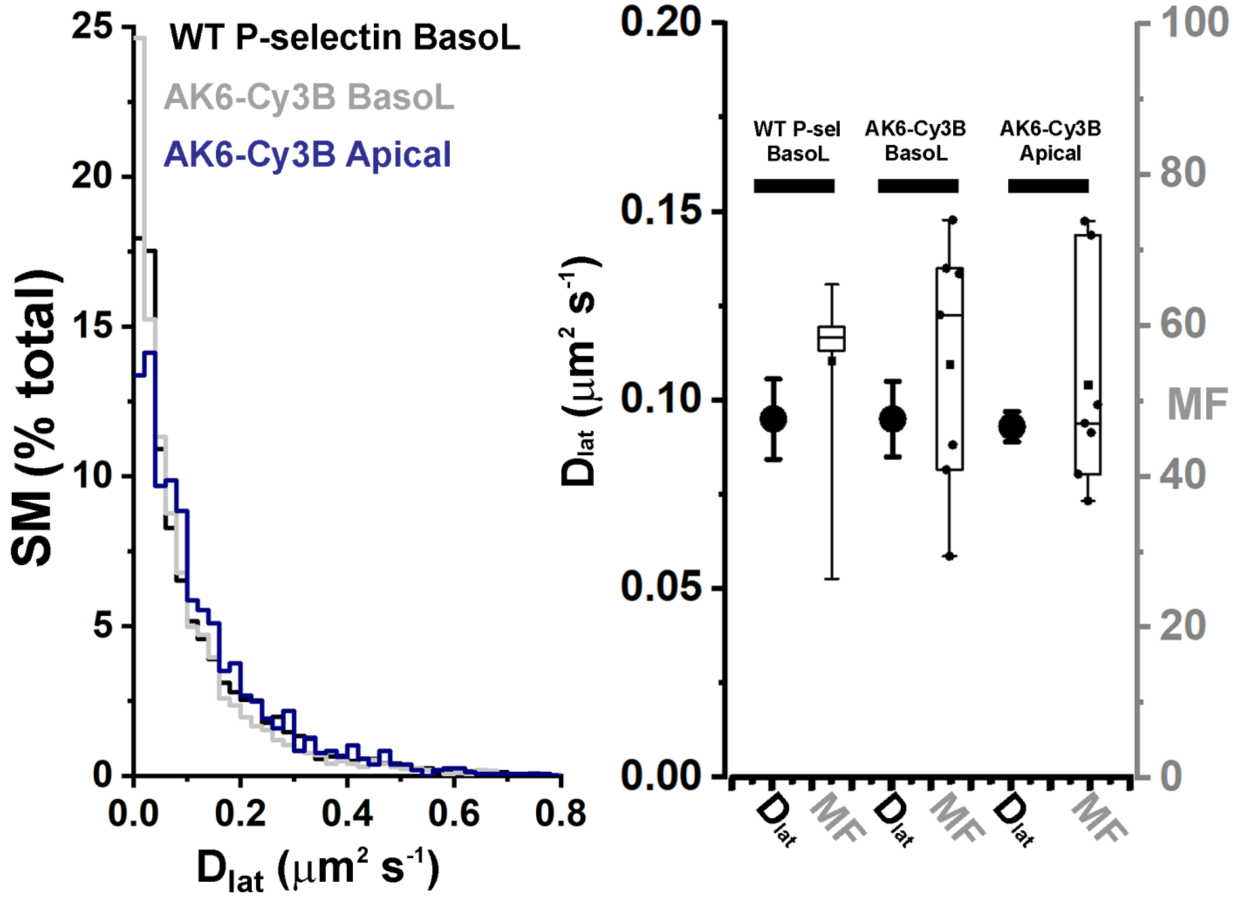

Supplementary Figure 6. P-selectin mobility is the same on both the apical and basolateral plasma membrane of HUVEC. Left; the distribution of D_{lat} for AK6-Cy3B on the apical plasma membrane (blue trace; 1,376 SMs, n= 6 cells, 2 experiments) or basal plasma membrane (that is in contact with the glass coverslip) (black; data duplicated across from Supplementary Figure 5). For comparison data for WT P-selectin-eGFP is duplicated across from Figure 1 of main text. Right; summary of D_{lat} and MF for WT P-selectin-eGFP and AK6-Cy3B on the basal plasma membrane, and AK6-Cy3B on the apical plasma membrane: Solid symbols are non-linear least-squares fitted D_{lat} values with 95% confidence limits shown as error bars; MF Boxes = 25%-75% intervals, horizontal bar = median, solid square = mean, Whiskers = 5%-95% intervals (all data values shown as dots).

.

Supplementary Figure 7

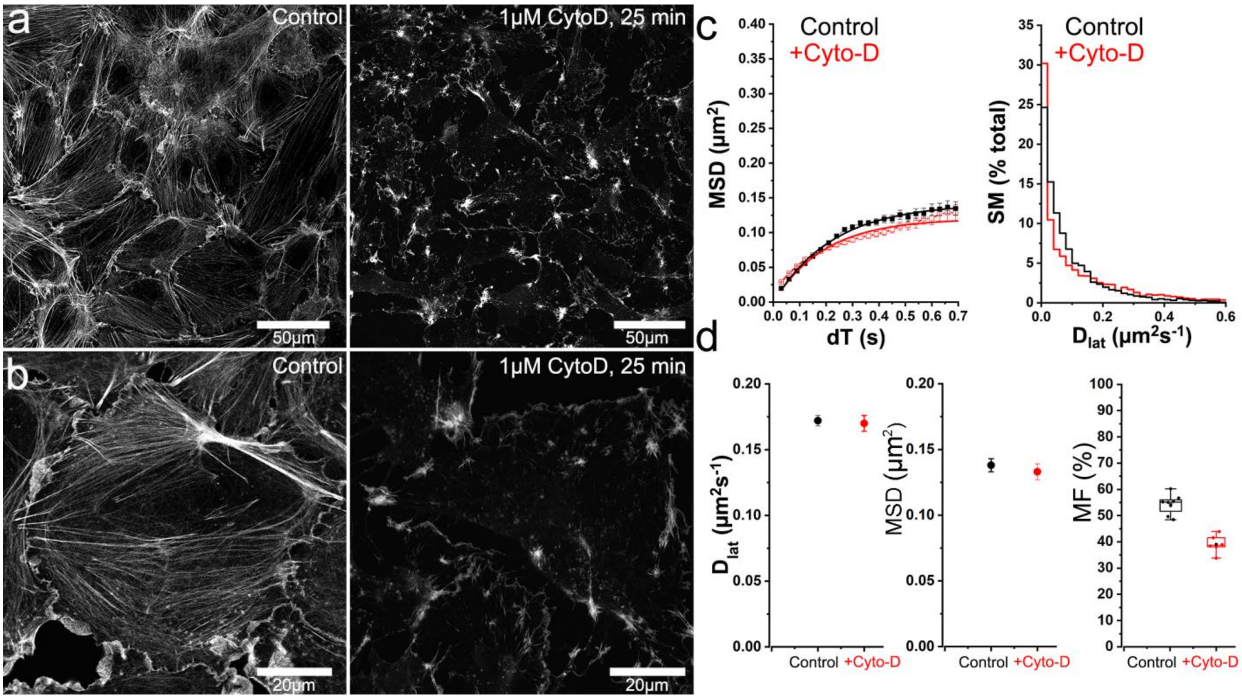

<u>Supplementary Figure 7.</u> Actin disruption has little effect on P-selectin mobility at steady state. **a-b**) shows confocal images of fixed HUVEC labeled with rhodamine-phalloidin in control (left) and cytochalasin-D (1 μM, for 25 min) and low (**a**) and high magnification (**b**). Images are representative of 3 separate experiments. Imaged using a Bio-Rad Radiance E2100 laser scanning confocal system, 60x 1.4NA. **c**) *MSD* v *dT* plot (left) and steady-state distributions of D_{lat} (right) for WT P-selectin-eGFP in the absence (black, 3,012 SFs, n=8 cells, 3 experiments) or after cytochalasin-D treatment (red, 5,762 SFs, n=6 cells, 3 experiments). The graph symbols are as described for Supplementary Figure 2. **d**) Summary graphs for control and cytochalasin-D treated conditions showing: non-linear least-squares fitted values for D_{lat} and limiting *MSD* ($\overline{MSD}$) (with 95% confidence intervals shown) and mobile fraction (MF) where: Boxes = 25%-75% intervals, horizontal bar = median, solid square = mean, Whiskers = 5%-95% intervals (all data values shown as dots).

<u>Supplementary Figure 8</u>

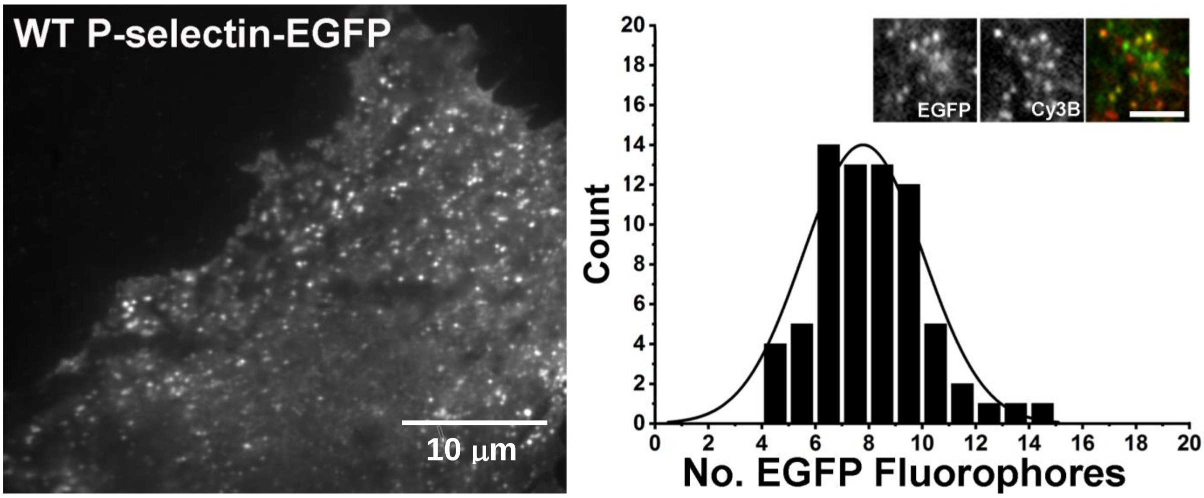

<u>Supplementary Figure 8.</u> Immobile clusters of P-selectin-eGFP on the plasma membrane of HUVEC. Left) TIRFM image (10 frame average) of the footprint of live P-selectin-eGFP expressing HUVEC at steady-state showing immobile bright spots of eGFP fluorescence on the plasma membrane. **Right**) Distribution of fluorescence intensities relative to the mean fluorescence of a single fluorophore (SF) for immobile spots (mean 7.8±0.26 SM, s.e.m., 75 spots, n=6 cells). Images inset; cells were briefly labeling with AK6-Cy3B (red in merged image) to confirm the cell surface localization of P-selectin-eGFP clusters (green in merged image). Scale bar 2 µm.

Supplementary Figure 9

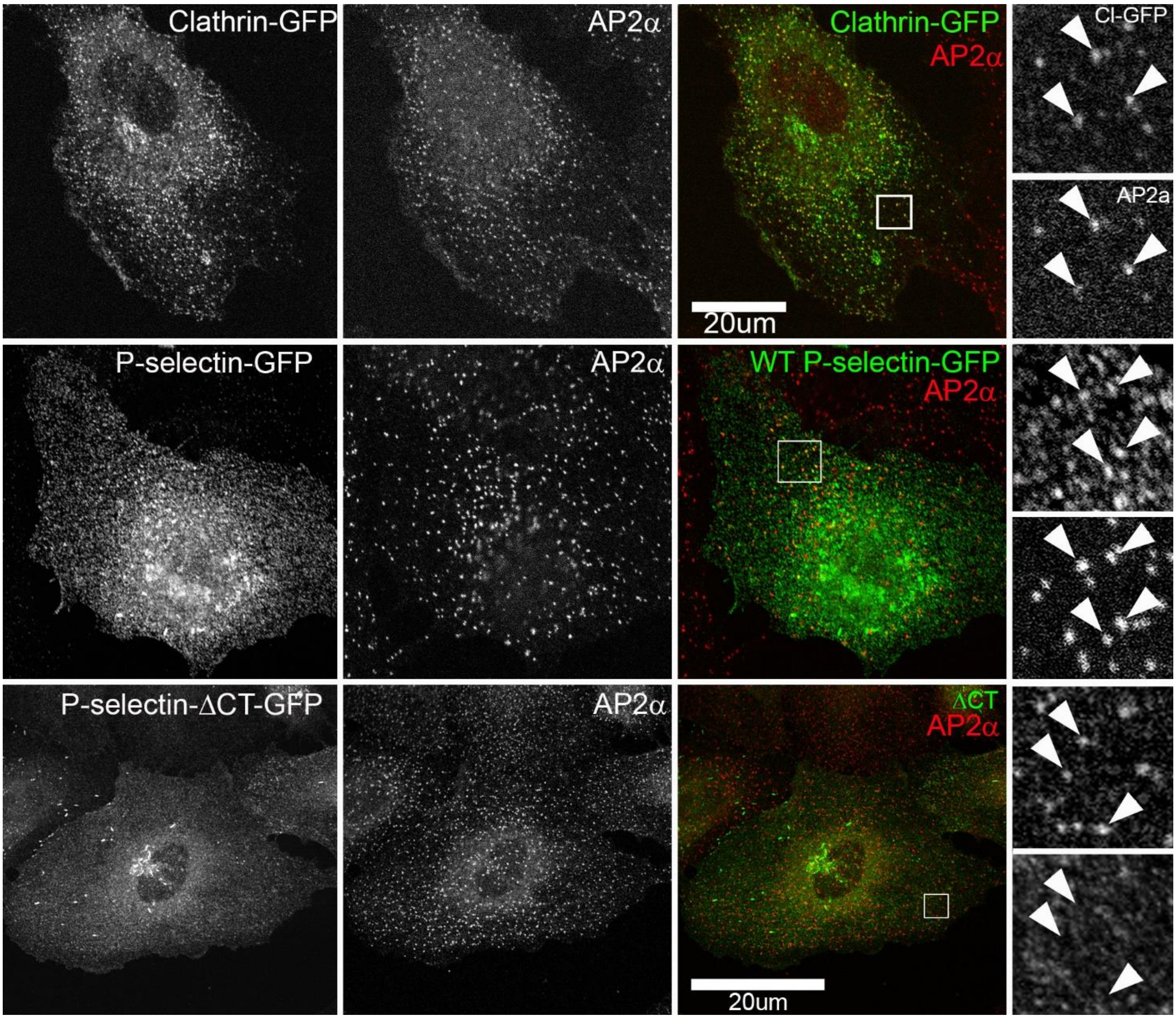

Supplementary Figure 9. P-selectin localizes with AP2 in a CT-dependent fashion. Confocal fluorescence images of fixed HUVEC 16-24 h after nucleofection with either clathrin-eGFP (Top left), WT P-selectin-eGFP (middle left) or P-selectin-ΔCT-eGFP (bottom left), and green in color merge images. In each case, cells were immuno-labeled for the endogenous AP2α subunit (middle panels and red in color merge images). Regions indicated by the white boxes are shown to the right in grey scale, and show co-localization of clathrin and AP2α (top), WT P-selectin with endogenous AP2α subunit (middle) but no co-localization of P-selectin-ΔCT-eGFP with endogenous AP2α (bottom). All images shown are representative of 2 independent experiments. Scale as indicated.

Supplementary Figure 10

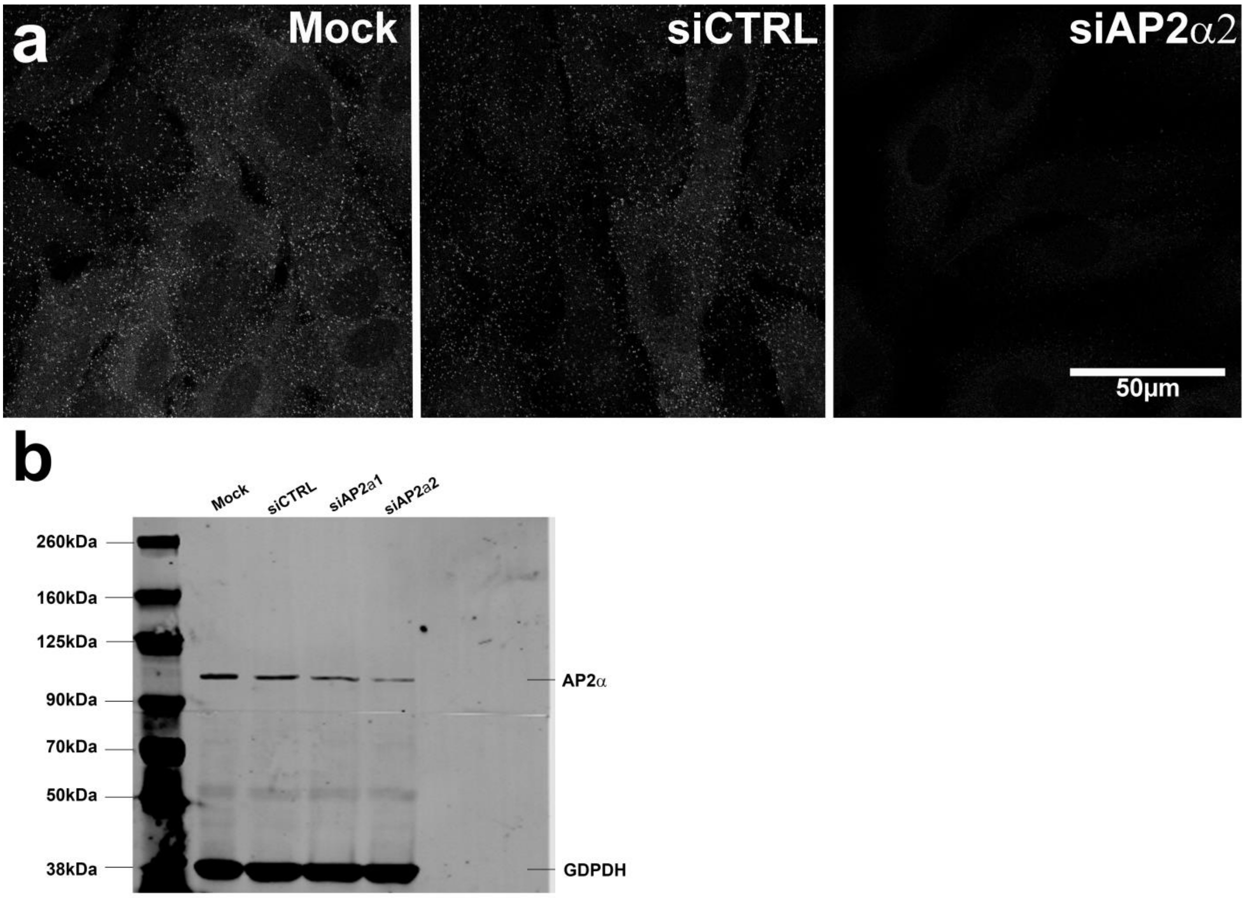

Supplementary Figure 10. siRNA mediated disruption of the AP2 complex. a) immuno-staining for the endogenous AP2α subunit in HUVEC following two rounds of Mock transfection (left; transfection with siRNA buffer alone), transfection with Dharmacon on-target-plus siControl oligonucleotides (200 pMol; Discovery Biosciences Limited UK catalog number: D-001810-10) or siAP2α2 (200 pMol; AAGAGCATGTGCACGCTGGCCA, Horizon Discovery Biosciences Limited UK) as described in Main Text Methods. Images are representative of 2 separate experiments. **b**) Western blot for endogenous Ap2α in Mock, siControl, siAP2α1 and siAP2α2 treated cells. Quantification of protein depletion for siAP2α2 normalized to siCTRL, 32% and 44% (n=2). Protein depletion for siAP2α1 (200pMol; GCATGTGCACGCTGGCCA, Horizon Discovery Biosciences Limited UK) was less effective and was not analyzed further.

Supplementary Figure 11

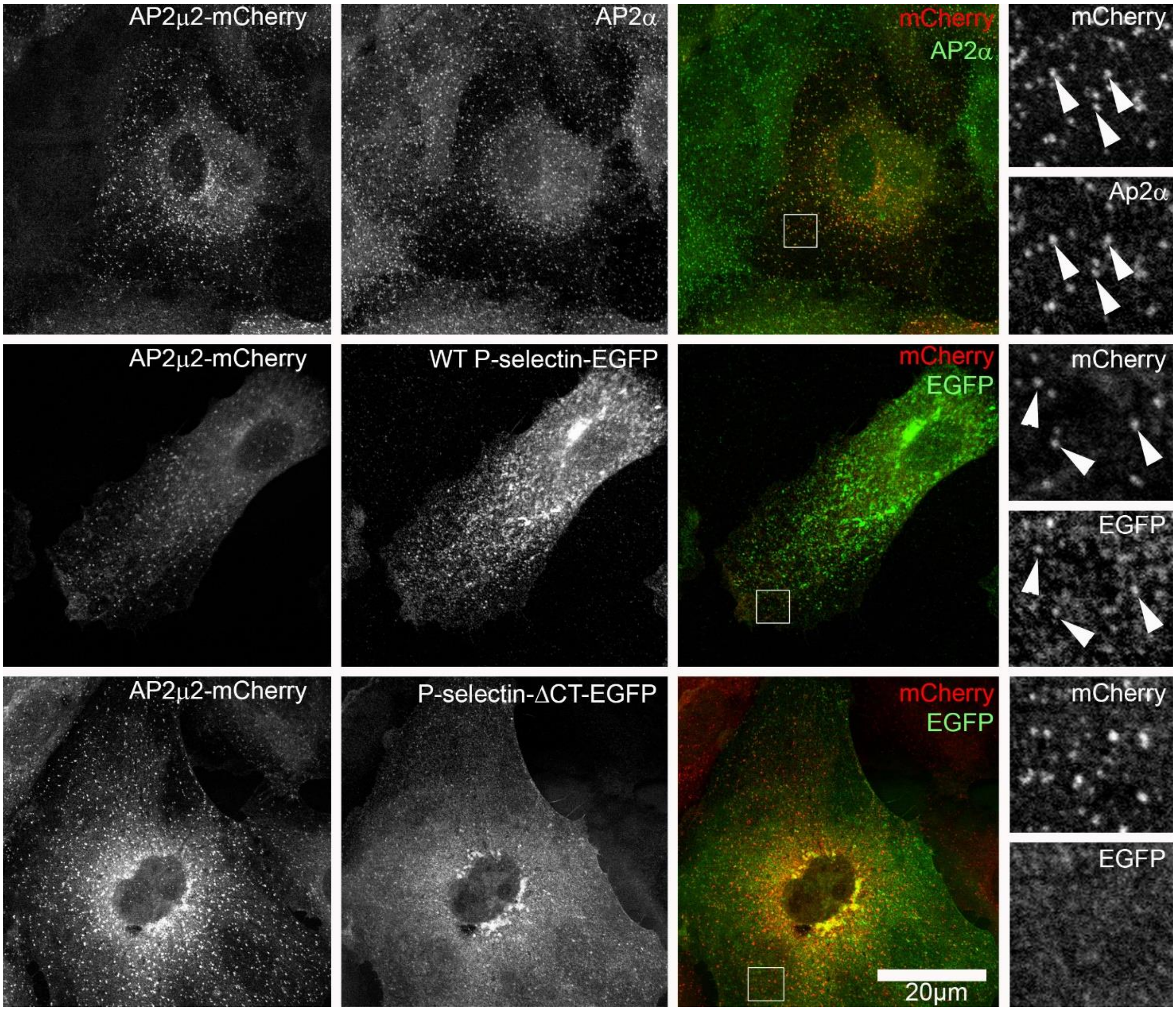

Supplementary Figure 11 P-selectin localizes with the AP2µ2 subunit in a CT-dependent fashion. Confocal fluorescence images of fixed HUVEC 16-24 hours after nucleofection with AP2µ2-mcherry (left panels and red in color merge) and either immuno-stained for the endogenous AP2α (top middle), or co-expressing WT P-selectin-eGFP (middle middle) or P-selectin-ΔCT-eGFP (bottom middle), green in color merge images. Regions indicated by the white boxes are shown to the right in grey scale, and show co-localization of AP2µ2-mcherry with endogenous AP2α (top), WT P-selectin (middle) but no co-localization with P-selectin-ΔCT-eGFP (bottom). All images are representative of 2 separate experiments. Scale bars as indicated.

Supplementary Figure 12

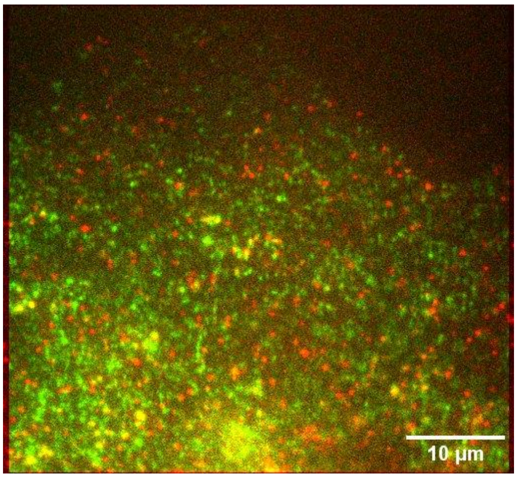

First frame (50ms) of video

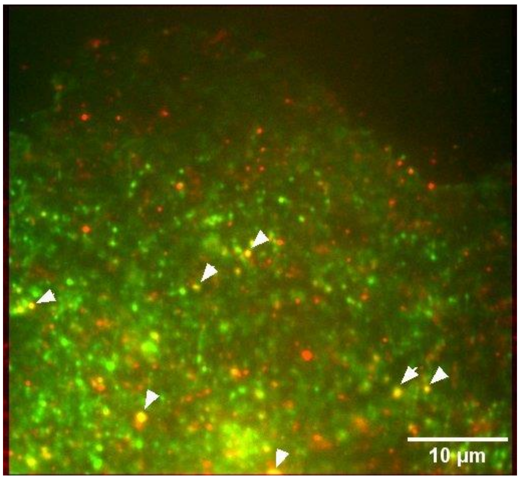

Average of 50 frames (2.5 s)

**Single particle tracking**	Average	Stdev	nCells	nTracks CD63	nTracks P-sel
Static track pairs (%)	**3.4%**	+/-2.7%	5		
Mobile pairs (%)	**0.50%**	+/-0.4%	5		
Total Tracks:				2853	1991
**Cross-correlation analysis**					
Static fraction Cross-correlation (%)	**5.1%**	+/-2.1%	5		
Mobile fraction Cross-correlation (%)	**0.74%**	+/-0.9%	5		

Supplementary Figure 12. Co-localization of CD63 and P-selectin in live HUVECs: Dual-color TIRF imaging and single particle tacking was performed in live HUVECs transfected with eGFP-CD63 (**green** signal) and later incubated with AK6-Cy3B (**red**) labelled P-selectin (see Methods in Main Text). Upper panels (left) shows the first frame (50 ms exposure) of a video sequence, in which moving single fluorophores are captured as diffraction-limited spots while clusters are brighter (some appear larger due to the display lookup table clipping artifact). The right image is an average of 50 frames (2.5 s) note that moving molecules are now obscured by motion blurring and static objects become more obvious. Tracking analysis showed that the vast majority of both P-selectin and CD63 molecules moved freely and separately at the membrane but static clusters were observed (arrows), and many contained multiple fluorophores of both colors. Lower Panel is a table showing that co-labelled static clusters comprised 3.4% of the total objects tracked and 5.1% of the total cross-correlated intensity (see Supplementary Figure 5 for more detail on how tracking and coincident tracks were defined). Only ~0.5% of all moving objects (defined as having $D_{lat} > 0.05\ \mu m^2.s^{-1}$) showed coincident or "paired" track trajectories. An important consideration is that wild-type HUVECs used in this study will contain a native pool of CD63 and P-selectin. These unlabeled (i.e. invisible) proteins would compete with the labelled species, so, our estimates of hetero-dimerization must be considered lower bounds. The full data set and *ImageJ* macro script used to perform cross-correlation analysis are provided in the manuscript source data file.

Supplementary Figure 13

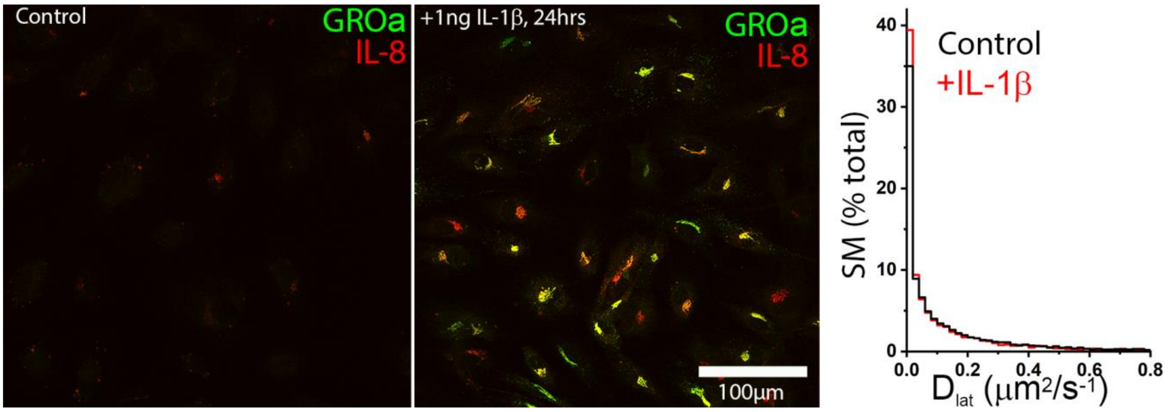

Supplementary Figure 13. Steady state Mobility of P-selectin is not changed in IL-1β treated HUVEC. Left) Confocal fluorescence images of fixed HUVEC 24 hours after treatment with vehicle or 1 ng.ml^{-1} IL-1ß, and immuno-stained with specific antibodies to endogenous IL-8 (red) and GROα (green). **Right**) Steady-state distributions of D_{lat} for WT P-selectin-eGFP in the absence (black, 15,280 SFs, n=14 cells, 2 independent experiments) or after IL-1β treatment (red, 10,214 SFs, n=10 cells, 2 independent experiments).

Supplementary References

1. Mashanov, G.I., Tacon, D., Knight, A.E., Peckham, M. & Molloy, J.E. Visualizing single molecules inside living cells using total internal reflection fluorescence microscopy. *Methods* **29**, 142-152 (2003).
2. Mashanov G.I. & Molloy J.E. Automatic detection of single fluorophores in live cells. *Biophys J* **92**, 2199-2211 (2007).
3. Mashanov G.I. Single molecule dynamics in a virtual cell: a three-dimensional model that produces simulated fluorescence video-imaging data. *J Roy Soc Interface* **11**, 20140442 (2014).
