# Supplementary material for: P-selectin mobility undergoes a sol-gel transition as it diffuses from exocytosis sites into the cell membrane
Source: Nat Commun. 2022 May 31;13:3031. doi: 10.1038/s41467-022-30669-x (PMC9156680; doi:10.1038/s41467-022-30669-x)
Supplement: Supplementary file 3 — Description of Additional Supplementary Files [file 41467_2022_30669_MOESM3_ESM.pdf]

File name: Supplementary Movie 1

Description: **Movie showing individual eGFP-Pselectin molecules released by histamine-evoked WPB exocytosis.** Regions of interest in TIRFM video recordings, centered on individual WPBs, were concatenated so that multiple secretion events could be visualized and tracked. Each trajectory was color-coded according to its lateral diffusion coefficient (D_{lat}) determined from the initial gradient of MSD vs dT plots, and all trajectories were then overlaid in a single image as a pseudo-color heat-map.
