# Supplementary material for: P-selectin mobility undergoes a sol-gel transition as it diffuses from exocytosis sites into the cell membrane
Source: Nat Commun. 2022 May 31;13:3031. doi: 10.1038/s41467-022-30669-x (PMC9156680; doi:10.1038/s41467-022-30669-x)
Supplement: Supplementary file 5 — Reporting Summary [file 41467_2022_30669_MOESM5_ESM.pdf]

Corresponding author(s): Tom Carter
Justin Molloy

Last updated by author(s): Apr 19, 2022

Reporting Summary

Nature Research wishes to improve the reproducibility of the work that we publish. This form provides structure for consistency and transparency in reporting. For further information on Nature Research policies, see our Editorial Policies and the Editorial Policy Checklist.

Statistics

For all statistical analyses, confirm that the following items are present in the figure legend, table legend, main text, or Methods section.

n/a	Confirmed	
☐	☒	The exact sample size (*n*) for each experimental group/condition, given as a discrete number and unit of measurement
☐	☒	A statement on whether measurements were taken from distinct samples or whether the same sample was measured repeatedly
☐	☒	The statistical test(s) used AND whether they are one- or two-sided<br>*Only common tests should be described solely by name; describe more complex techniques in the Methods section.*
☒	☐	A description of all covariates tested
☒	☐	A description of any assumptions or corrections, such as tests of normality and adjustment for multiple comparisons
☐	☒	A full description of the statistical parameters including central tendency (e.g. means) or other basic estimates (e.g. regression coefficient) AND variation (e.g. standard deviation) or associated estimates of uncertainty (e.g. confidence intervals)
☐	☒	For null hypothesis testing, the test statistic (e.g. *F*, *t*, *r*) with confidence intervals, effect sizes, degrees of freedom and *P* value noted<br>*Give P values as exact values whenever suitable.*
☒	☐	For Bayesian analysis, information on the choice of priors and Markov chain Monte Carlo settings
☒	☐	For hierarchical and complex designs, identification of the appropriate level for tests and full reporting of outcomes
☒	☐	Estimates of effect sizes (e.g. Cohen's *d*, Pearson's *r*), indicating how they were calculated

Our web collection on statistics for biologists contains articles on many of the points above.

Software and code

Policy information about availability of computer code

Data collection

Data collection was via a custom-built microscope system. The data collection software is device-specific but it is freely available (at the users risk) on request.

Data analysis

Image analysis was performed using custom software, "GMimPro", that is freely available (www.mashanov.uk), has been reported in other publications and has been downloaded and used many times by other research groups. Other data analysis was performed in Excel and IgorPro (as referenced in main text) and in GraphPad Prism (referenced in main text and figure legends). The short ImageJ Macro used to perform Pearson cross-correlation of two-color single fluorophore movies (referred to Supplementary Information) is attached in the "Source Data" excel spreadsheet under Figure S12 tab.

Software Versions:
Adobe Photoshop CC, 345 Park Avenue, San Jose, CA, USA
GMimPro Ver.2022 64-bit. Freeware available at https://www.mashanov.uk (Gregory Mashanov, Francis Crick Institute, London NW1 1AT, UK. Email: gregory.mashanov@crick.ac.uk)
GraphPad Prism Vr 9.0.2 (2021), Northside Dr. San Diego, CA, USA
IgorPro 8.0, Wavemetrics, Lake Oswego, OR, USA.
ImageJ 1.53f http://imagej.nih.gov/ij
OriginLabs Origin 2018 (64 bit) SR1, One Roundhouse Plaza, Northampton, MA, USA
Microsoft Excel (2019) Solver add-in, GRG Non-linear, least squared error minimization (Frontline Systems, Inc. Incline Village, NV. USA)

For manuscripts utilizing custom algorithms or software that are central to the research but not yet described in published literature, software must be made available to editors and reviewers. We strongly encourage code deposition in a community repository (e.g. GitHub). See the Nature Research guidelines for submitting code & software for further information.

Data

Policy information about availability of data

All manuscripts must include a data availability statement. This statement should provide the following information, where applicable:

- Accession codes, unique identifiers, or web links for publicly available datasets
- A list of figures that have associated raw data
- A description of any restrictions on data availability

Source Data is provided in MS Excel format accompanying this manuscript available on-line at Nature Communications. The figures for which source data is available are: Main text Figures 1-6, Supplementary Figures 2, 4, 5-8, 10, 12, 13. An example video, Supplementary Movie 1 is provided on-line. We find compressed video formats (e.g. AVI, MP4, MOV, WMV, FLV etc) of mobile single molecules are of low quality as compression algorithms perform poorly with this type of video data. Raw video imaging data, consisting of many tens of gigabytes of separate video data files, are available from the corresponding authors upon reasonable request.

Field-specific reporting

Please select the one below that is the best fit for your research. If you are not sure, read the appropriate sections before making your selection.

☒ Life sciences ☐ Behavioural & social sciences ☐ Ecological, evolutionary & environmental sciences

For a reference copy of the document with all sections, see nature.com/documents/nr-reporting-summary-flat.pdf

Life sciences study design

All studies must disclose on these points even when the disclosure is negative.

Sample size

Number of molecules, number cells and experimental repeats were counted and reported in Figure Legends for each experimental condition. The error within each experiment (i.e. variation between molecules) is governed by statistical mechanics and the stochastic variation in the different data sets is either Gaussian or exponentially distributed depending on the type of measurement being made. The Gaussian variance depends on absolute temperature and system stiffness and various imaging parameters. The measurement bandwidth should sample the power spectral density of the statistical fluctuations. The variance in MSD vs dT plots and lifetime analysis has been described, studied and published many times. The refs 22-27 and 32&33 in the Main Text cover this topic. Variation in data sets between exocytosis events is dominated by stochastic noise (i.e. because <300 molecules can be tracked for each exocytosis event and we need >1000 to get "reasonable looking" MSD vs dT plots). Therefore, we sample many exocytosis events across many cells and several experimental repeats in order to accumulate sufficient data so that our stochastic variation (thermal statistical noise) is reduced and we can test for differences between the mutants. This is most obvious perhaps when we look at the untreated control cells vs. Heparanase, Chondroitinase and Neuraminidase experiment (i.e. plots in 6g&h). Sampling statistics for single molecule experiments has been dealt with extensively in the literature over the past 30 years and it is beyond the scope of the current paper and certainly this short Reporting Summary to delve deeply into the topic.

Data exclusions

The specific criteria set for identifying optical signals arising from individual molecules was reported in an earlier publication (cited in the paper by Mashanov et al, Methods 29, 142-152 (2003)). No data were otherwise excluded except for when there were obvious technical problems., including focus drift, material passing over the observation area during recording, sample movement etc...

Replication

samples were replicated by performing separate transfections, using different cells from different wells, and different cells from the same well.

Randomization

There is no observer bias in our data analysis because all downstream video analysis is performed automatically by computer tracking. I.e. the computer does not "know" what sample is being analyzed. Our analysis program (GMImPro, referenced above) generates all the summary plots and statistics and we validate our tracking results by visual inspecting of the output trajectories against the raw video data. The software only "goes wrong" for the reasons discussed above ("Data Exclusions") e.g. focus drift, stage movement, debris in the sample.

Blinding

As for "Randomization": Sample data are analyzed by computer using a fully automated image analysis program. The computer program is technically "blind" to the sample.

Reporting for specific materials, systems and methods

We require information from authors about some types of materials, experimental systems and methods used in many studies. Here, indicate whether each material, system or method listed is relevant to your study. If you are not sure if a list item applies to your research, read the appropriate section before selecting a response.

Materials & experimental systems

n/a	Involved in the study
☐	☒ Antibodies
☐	☒ Eukaryotic cell lines
☒	☐ Palaeontology and archaeology
☒	☐ Animals and other organisms
☒	☐ Human research participants
☒	☐ Clinical data
☒	☐ Dual use research of concern

Methods

n/a	Involved in the study
☒	☐ ChIP-seq
☒	☐ Flow cytometry
☒	☐ MRI-based neuroimaging

Antibodies

Antibodies used

These reagents are Tabulated in Supplementary Information and reported here for completeness (This PDF file does not support Table Formatting)
Antibody reagents
Species Manufacturer Clone / catalogue number / Dilutions used for ICC or WB
1) VWF Rabbit pAb DAKO/Agilent Santa Clara, CA USA A0082 1:10000
2) VWF Sheep pAb Serotec Raleigh, NC, USA AHP062 1:10000
3) P-selectin Mouse pAb Bio-Rad (formerly AbD Serotec) AK6/MCA796 1:50
4) Control mouse IgG Mouse pAb Invitrogen/ThermoFisher 16-4714-82 1:50
5) Heparan sulphate Rabbit mAb Sigma-Aldrich MAB1948P/A7L6 1:500
6) Chondroitin-4-sulphate Mouse mAb Sigma-Aldrich MAB2030/clone BE-123 1:50
7) FITC-WGA - Vector Labs Inc Burlingame, CA, USA FL-1021 1:400
8) anti-GFP Rabbit mAb Invitrogen/ThermoFisher A-6455 1:300
9) anti-GFP Sheep pAb Bio-Rad Labs Watford, UK 4745-1051 1:250
10) AP2alpha Mouse mAb Invitrogen/ThermoFisher AP6/ MA1-064 1:400
11) anti RFP Rabbit pAb Abcam Cambridge, UK ab62341 1:200
12) Rhodamine-phalloidin - Invitrogen/ThermoFisher R415 1:200
13) GROα Mouse MAb R&D systems Abingdon, UK MAB275 / clone 20326 1:100
14) IL-8 Goat pAb R&D systems AF-208-NA 1:100
15) Anti-alpha adaptin Mouse mAb BD Transduction labs 610502 1:1000 (WB)
16) GAPDH (control : 36kDa) Mouse mAb Merck Millipore MAB374, clone 6C5 1:5000 (WB)
17) Fluorophore coupled secondary antiobodies: various Jackson ImmunoResearch Europe (Newmarket, UK)
Alexa 488 AffiniPure Donkey Anti-Mouse IgG (715-545-150)
Rhodamine Red™-X (RRX) AffiniPure Donkey Anti-Mouse IgG (115-295-003)
Cy™5 AffiniPure Donkey Anti-Rabbit IgG (711-175-152)
Alexa 488 AffiniPure Donkey Anti-Rabbit IgG (711-545-152)
Rhodamine Red™-X (RRX) AffiniPure Donkey Anti-Rabbit IgG (711-295-152)
Alexa 488 AffiniPure Donkey Anti-Sheep IgG (713-545-147) 1:1000

Validation

As noted by manufacturers and as below (key numbers as in previous entry)
1. Anti-Human VWF (A0082) has been validated for detection of von Willebrand factor in endothelial cells, megakaryocytes and platelets when tested on formalin-fixed, paraffin-embedded normal human tissues (bone marrow, kidney, liver, lung, lymph node, skin and spleen) as described on the Agilent website https://www.agilent.com/en/product/dako-omnis-solution-for-ihc-ish/primary-antibodies-for-dako-omnis/primary-antibodies-(flex-ready-to-use)/von-willebrand-factor-(dako-omnis)-76216). We use the Ab extensively in our lab for ICC and WB and it recognises epitope tagged VWF both in fixed and live HUVEC.
2. Anti-human VWF Ab has been verified for detection of VWF by Serotec and in multiple publications as described on the BioRad website https://www.citeab.com/antibodies/111902-ahp062-sheep-anti-human-von-willebrand-factor). We have used the Ab extensively in our lab for ICC and WPB and it recognises epitope tagged VWF in fixed aHUVEC.
3. Anti-CD62P/P-selectin (AK6), Immunofluorescence; has been verified for detection of P-selectin by Serotec labs and in multiple publications as described on the BioRad website (https://www.bio-rad-antibodies.com/monoclonal/human-cd62p-antibody-ak-6-mca796.html?f=purified). In our hands AK6 recognises epitope tagged P-selectin expressed in HUVEC.
4. Control mouse IgG: This is commonly used as an isotype control reagent as described on the manufactures website (https://www.thermofisher.com/antibody/product/Mouse-IgG1-kappa-clone-P3-6-2-8-1-Isotype-Control/16-4714-82). Cy3b conjugated IgG was used on 3% paraformaldehyde fixed and live HUVEC and showed no labelling of cells when probed with a goat anti-mouse secondary Ab from Jackson ImmunoResearch.
5. Anti-Heparan sulphate Ab (MAB1948P) this Anti-Heparan Sulfate Proteoglycan (Perlecan) Antibody, clone A7L6, is validated for use in IH, IC, WB & IP according the manufactures web site https://www.merckmillipore.com/GB/en/product/Anti-Heparan-Sulfate-Proteoglycan-Perlecan-Antibody-clone-A7L6,MM_NF-MAB1948P .
6. Anti-Chondroitin-4-sulphate (MAB2030): Immunofluorescence; this antibody only reacts with digested material. Fixed tissue must be digested with chondroitinase ABC (Reine, TM., et al., PubMed ID: 23757342). No reaction with native proteoglycans or with proteoglycans digested with either chondroitinase AC or testicular hyaluronidase. Reacts with both mouse (fetal and adult) and human (fetal and adult) chondroitinase ABC digested proteoglycans. https://www.sigmaaldrich.com/GB/en/product/mm/mab2030 .
7. FITC-WGA (FL-1021): Immunofluorescence; detects N-Acetylglucosamine, positive staining validated by pre-blocking the lectin with its inhibiting sugar Chitin Hydrolysate. Used in >130 publications according to manufactures website https://vectorlabs.com/products/glycobiology/fluorescein-wheat-germ-agglutinin-wga#biozbadges.
8. Anti-GFP rabbit (A-6455): Immunofluorescence, WB. Validated by WB in HEK293 cells expressing GFP (manufactures website). Detected GFP-tagged P-selectin expressed in HUVEC. More than 120 publications using the Ab for detection of GFP by

Immunofluorescence (https://www.thermofisher.com/antibody/product/GFP-Antibody-Polyclonal/A-6455)
9. Anti-GFP sheep (4751-1051): Immunofluorescence, this product has been reported to work by Immunofluorescence from testing within Serotec laboratories, and in multiple peer-reviewed publications, as described on the suppliers web site https://www.bio-rad-antibodies.com/polyclonal/green-fluorescent-protein-antibody-4745-1051.html?f=purified. In our hands it recognised GFP-tagged P-selectin expressed in HUVEC.
10. Anti-AP2alpha, MA1-064. Detects assembly polypeptide 2 (AP2) from a wide variety of mammalian sources, including human, hamster, monkey, bovine, rat, and mouse. It recognizes the products of both alpha-adaptin genes, alpha A and alpha C as well as an alternatively spliced isoform of alpha adaptin found in neurons. MA1-064 has been successfully used in Western blot, immunofluorescence, immunocytochemistry, and immunoprecipitation procedures. By Western blot, this antibody detects an ~100 kDa doublet representing the two isoforms of alpha adaptin in rat brain (alpha C is the dominant form seen).
11. Anti-RFP antibody : ab62341 recognizes RFP and has been shown to react with tdTomato, It is validated for ICC and a wide range of other techniques. https://www.abcam.com/rfp-antibody-ab62341.html?productWallTab=Abreviews&applications=3688&PageSize=10&SortOrder=VoteDesc .
12. Rhodamine-phalloidin (R415). Rhodamine phalloidin is one of the most commonly used fluorescent phalloidin conjugates in the literature, as evidenced by over 1,500 citations https://www.thermofisher.com/order/catalog/product/R415
13. Anti-GROα (MB275). Validated for immunofluorescence in our laboratory as described in Knipe, L. et al. Blood 116, 2183-2191 (2010).
14. Anti-IL-8 (AF-208-NA). Validated for immunofluorescence by the manufacturer and used successfully in our laboratory as described in Knipe, L. et al. Blood 116, 2183-2191 (2010).
15. Anti-alpha adaptin (PA5-17029). This antibody is validated and routinely tested by Western blot by the manufacturer as described on their web pages https://www.bdbiosciences.com/ja-jp/products/reagents/microscopy-imaging-reagents/immunofluorescence-reagents/purified-mouse-anti-adaptin.610502
16. Anti-GAPDH: clone 6C5 is a well published and extensively characterized monoclonal antibody. This purified mAb detects Glyceraldehyde-3-Phosphate Dehydrogenase (GAPDH) & has been published & validated for use in ELISA, IP, IC, IF, IH & WB.
17. Fluorophore-coupled secondary antibodies: These are well-established lab reagents principally validated by the same methods as primary Abs as well as negative controls lacking primary antibodies (see e.g. above). They have been independently validated by the manufacturer, our own laboratories and many labs around the world.

Eukaryotic cell lines

Policy information about cell lines

Cell line source(s)	We do not use cell lines, we use pooled primary Human Umbilical Vein Endothelial Cells (HUVECs) (PromoCell GmbH, Heidelberg, Germany: catalog C12203). Cell were cultured (maximum passage, P4) in Medium 199 Earle's salts + L-Glutamine (Catalog 11150059, ThermoFisher), supplemented with 20% fetal calf serum, 30 µg/ml Endothelial Cell Growth Supplement, 10 U/ml heparin and 50 µg/ml gentamicin at 37oC in a 5% CO2 atmosphere.
Authentication	These primary cells express HUVEC specific proteins (P-selectin, CD63, VWF) and structures (WPB) and are validated by the supplier. All cells had abundant Weibel Palade Bodies, clearly visible by optical microscopy and which exocytosed in response to histamine stimulation.
Mycoplasma contamination	Cells were tested in-house for mycoplasma contamination.
Commonly misidentified lines (See ICLAC register)	None.
